# Stabilization by Fusion to the C-terminus of Hyperthermophile *Sulfolobus tokodaii* RNase HI: A Possibility of Protein Stabilization Tag

**DOI:** 10.1371/journal.pone.0016226

**Published:** 2011-01-19

**Authors:** Kazufumi Takano, Tomohiro Okamoto, Jun Okada, Shun-ichi Tanaka, Clement Angkawidjaja, Yuichi Koga, Shigenori Kanaya

**Affiliations:** 1 Department of Material and Life Science, Osaka University, Osaka, Japan; 2 Core Research for Evolutional Science and Technology (CREST), Japan Science and Technology Agency (JST), Osaka, Japan; University of Oulu, Germany

## Abstract

RNase HI from the hyperthermophile *Sulfolobus tokodaii* (Sto-RNase HI) is stabilized by its C-terminal residues. In this work, the stabilization effect of the Sto-RNase HI C-terminal residues was investigated in detail by thermodynamic measurements of the stability of variants lacking the disulfide bond (C58/145A), or the six C-terminal residues (ΔC6) and by structural analysis of ΔC6. The results showed that the C-terminal does not affect overall structure and stabilization is caused by local interactions of the C-terminal, suggesting that the C-terminal residues could be used as a “stabilization tag.” The Sto-RNase HI C-terminal residues (-IGCIILT) were introduced as a tag on three proteins. Each chimeric protein was more stable than its wild-type protein. These results suggested the possibility of a simple stabilization technique using a stabilization tag such as Sto-RNase HI C-terminal residues.

## Introduction

An important goal of protein engineering is designing variants that enhance the conformational stability of proteins. Structure-based design [1], [2], sequence alignment approaches [3], [4], and random mutagenesis [5], [6] are all used to stabilize proteins by mutagenesis, but problems still exist, especially in finding simple and general techniques.

Protein tags, which are peptide sequences genetically grafted onto the N- or C-terminus of recombinant proteins, are widely used experimentally because they are easy to manipulate. Tags are attached to proteins for various purposes, such as purification [7], [8], solubilization [9], [10], and fluorescent imaging [11], [12]. Development of a “stabilization tag” will allow researchers to work with designed variants with enhanced protein stability.

**Figure 1:**
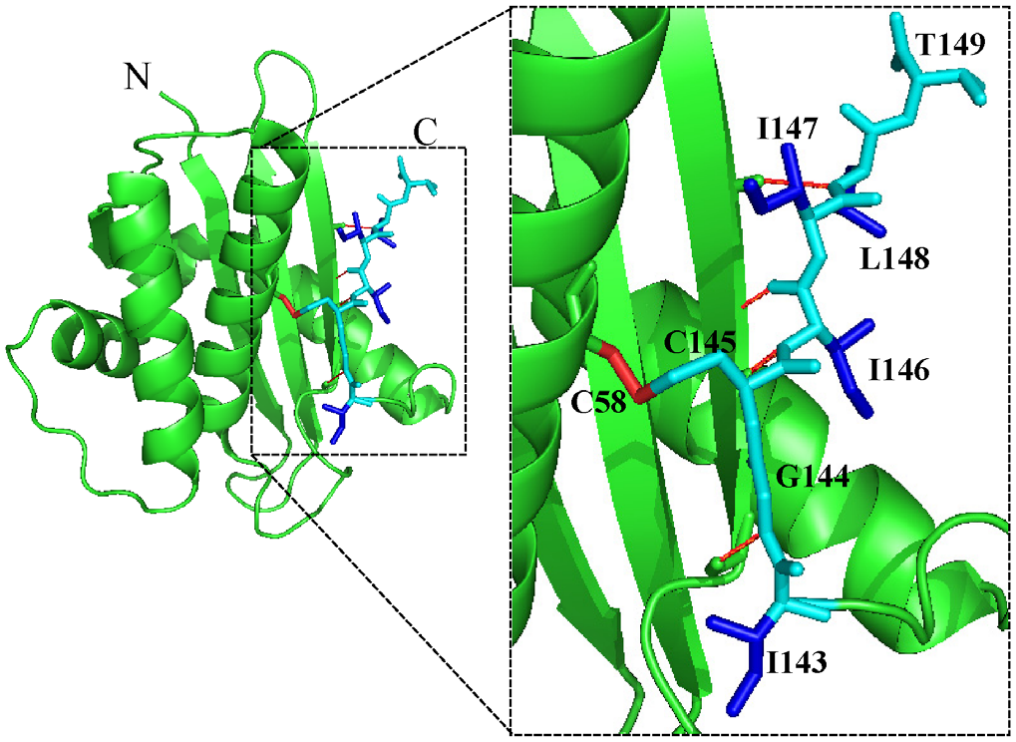
Crystal structure of wild-type Sto-RNase HI. C-terminal seven residues (cyan); hydrophobic side-chains (blue); hydrogen bonds (thin red lines); and disulfide bond (thick red line).

Ribonuclease HI from the hyperthermophile *Sulfolobus tokodaii* (Sto-RNase HI) is a monomeric protein of 149 amino acids [13], [14]. Sto-RNase HI is highly stable, and is stabilized through the C-terminal tail [14], [15]. The C-terminus of Sto-RNase HI is anchored to the core region by one disulfide bond (Cys58-Cys145), several hydrogen bonds, and hydrophobic interactions (Figure 1). Since the C-terminus of proteins is generally flexible, C-terminal anchoring may be useful for stabilization factors.

In this work, we analyzed stabilization by the amino acid residues of the Sto-RNase HI C-terminus, and aimed to develop a protein stabilization tag. First, we investigated in detail the stabilization effect of the Sto-RNase HI C-terminus, by measuring the stability of a C58/145A variant lacking the disulfide bond, and a ΔC6 variant lacking the six C-terminal residues. The Sto-RNase HI C-terminal residues IGCIILT were introduced onto three proteins as a tag, and the effect on stability was examined. We propose a stabilization tag as a novel protein stabilization technique. In future studies, we hope to design a universal stabilization tag or optimal stabilization tags for individual proteins.

## Methods

### Protein purification

Wild-type, C58/145A and ΔC6 Sto-RNase HI were overproduced and purified as previously described [13], [14]. RNase HI from the psychrotrophic bacterium *Shewanella oneidensis* MR-1 (So-RNase HI) [16], or from *Escherichia coli* (Ec-RNase HI) [17], and esterase from *Sulfolobus tokodaii* (Sto-esterase) [Angkawidjaja et al., in preparation] were overproduced and purified as described. Plasmids for the overexpression of variants of So-RNase HI, Ec-RNase HI, and Sto-esterase were constructed from the wild-type genes using standard recombinant DNA techniques. Overproduction and purification of the chimeric proteins was as for the wild-type proteins. Protein purity was confirmed using SDS-PAGE.

### Biophysical analyses

Circular dichroism (CD) spectra measurements, equilibrium and kinetic guanidine hydrochloride (GdnHCl)-induced, and heat-induced unfolding were as previously described [15]. Buffers were 20 mM glycine-HCl pH 3.0 for Sto-RNase HI, 10 mM acetate pH 5.5 with 1 M GdnHCl for So-RNase HI, 10 mM glycine-HCl pH 3.0 for Ec-RNase HI, and 25 mM CHES-NaOH pH 9.0 with 1 M GdnHCl for Sto-esterase.

Equilibrium experiments on GdnHCl-induced unfolding were examined by monitoring the CD at 220 nm. Protein solutions were incubated in GdnHCl at different concentrations and at different temperatures for unfolding. The GdnHCl-induced unfolding curves were determined, and a nonlinear least-squares analysis was used to fit the data to
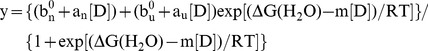
(1)

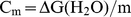
(2)where y is the observed CD signal at a given concentration of GdnHCl, [D] is the concentration of GdnHCl, b^0^_n_ is the CD signal for the native state, b^0^_u_ is the CD signal for the unfolded states, a_n_ is the slope of the pre-transition of the baseline, and a_u_ is the slope of the posttransition of the baseline. ΔG(H_2_O) is the Gibbs energy change (ΔG) of the unfolding in the absence of GdnHCl, m is the slope of the linear correlation between ΔG and the GdnHCl concentration [D], and C_m_ is the GdnHCl concentration at the midpoint of the curve. Two or three replicates were measured for each condition. The raw experiment data were directly fitted to Eq. (1) using SigmaPlot (Jandel Scientific).

Stability profiles (temperature dependence of ΔG(H_2_O)) were fitted to the Gibbs-Helmholtz equation, Eq. (3).
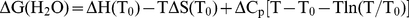
(3)where ΔH(T_o_) and ΔS(T_o_) are the enthalpy and entropy of unfolding at the reference temperature T_o_, and ΔC_p_ is the difference in heat capacity between the native and unfolded states.

Kinetic experiments on GdnHCl-induced unfolding were followed by CD spectra measurement at 220 nm. The unfolding reactions of proteins were induced by a concentration jump in GdnHCl, with various differing concentrations. The kinetic data were analyzed using Eq. (4).
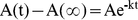
(4)


Here, A(t) is the value of the CD signal at a given time t, A(∞) is the value when no further change is observed, k is the apparent rate constant, and A is the amplitude. Two or three replicates were measured for each condition. The GdnHCl concentration dependence of the logarithms of the apparent rate constant (k_app_) for unfolding was also examined. The rate constants for unfolding in the absence of GdnHCl (k_u_(H_2_O)) were calculated by fitting to Eq. (5):
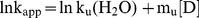
(5)where [D] is the concentration of GdnHCl and m_u_ represents the slopes of the linear correlations of ln k_u_ with the GdnHCl concentration.

Heat-induced unfolding was examined by monitoring the CD at 220 nm. All experiments were carried out at a scan rate of 1°C min^−1^. A nonlinear least-squares analysis was used to fit the data to
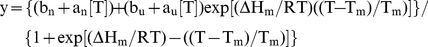
(6)where y is the observed CD signal at a given temperature [T], b_n_ is the CD signal for the native state, b_u_ is the CD signal for the unfolded states, a_n_ is the slope of the pretransition of the baseline, a_u_ is the slope of the posttransition of the baseline, ΔH_m_ is the enthalpy of unfolding at the transition midpoint temperature (T_m_), T is the temperature, and R is the gas constant. Curve fitting was performed using SigmaPlot. Two or three replicates were measured for each condition.

### Structural analysis

Crystals of ΔC6 Sto-RNase HI were grown in 20% PEG 3000, 0.1 M citrate pH 5.5, including 6–7 mg mL^−1^ protein at 4°C. All full diffraction sets were collected at 100 K without cryoprotectants on a SPring-8 BL38B1. Diffraction data were indexed, integrated, and scaled using the HKL2000 program suite [18]. The crystal structure was solved by the molecular replacement method using MOLREP [19] in the CCP4 program suite [20], with the wild-type structure (2EHG) as a starting model. Structure refinement was with the programs Coot and REFMAC in the CCP4 program suite [21], [22]. Progress in structure refinement was evaluated at each stage by the free R-factor and by inspecting stereochemical parameters calculated by the program PROCHECK [23]. Collected and refined data are in Table 1. Figures were prepared using PyMol (http://www.pymol.org).

**Table 1:** Statistics on data processing and structure determination of ΔC6 Sto-RNase HI.

Wavelength (Å)	1.0
Temperature (K)	100
Space group	P1
Unit cell edges (Å)	40.98, 41.02, 43.53
Unit cell angles (Å)	89.72, 89.71, 86.76
Resolution (Å)	50.0–1.66 (1.72–1.66)
No. measured reflections	244,376
No. unique reflections	32,375
R_merge_ (%)^a^	3.4 (21.8)
Completeness (%)	96.2 (94.3)
<I>/<σI>	37.2 (6.1)
R_work_/R_free_ (%)^b^	19.7/25.0
Total atoms (protein/solvent)	2244/256
Root-mean-square deviation	
Bond length (Å)	0.011
Bond angles (deg.)	1.391
Ramachandran plot	
Most favored (%)	94.6
Additionally allowed (%)	5.4
Disallowed (%)	0

Values in parentheses are the highest-resolution bin of respective data.

a R_merge_ = Σ |I_hkl_ - <I_hkl_>|/Σ I_hkl_, where I_hkl_ is the intensity measurement for reflection with indices hkl and <I_hkl_> is the mean intensity for multiply recorded reflections.

b R_work, free_ = Σ ||F_obs_| - |F_calc_||/Σ |F_obs_|, where the R-factors are calculated using the working and free reflection sets, respectively. The free reflections comprise a random 10% of the data held aside for unbiased cross-validation throughout refinement.

### Protein data bank accession number

The coordinates and structure factors of ΔC6 Sto-RNase HI have been deposited in the RCSB Protein Data Bank under ID code 3ALY.

## Results and Discussion

### CD spectra and crystal structure of Sto-RNase HI variants

**Figure 2:**
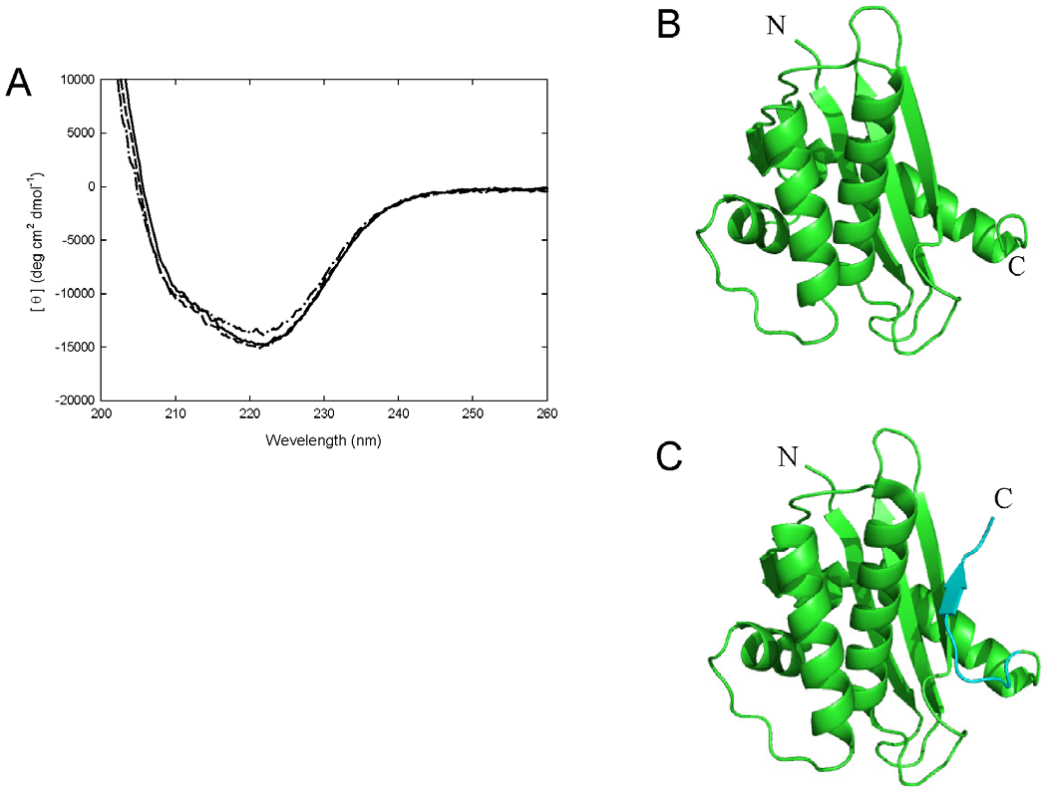
CD spectra and crystal structure of Sto-RNase HI variants. (A) CD spectra of wild-type (solid line), C58/145A (dashed line), and ΔC6 (dot-dashed line) Sto-RNase HI. (B) Crystal structure of ΔC6 Sto-RNase HI. (C) Crystal structure of wild-type Sto-RNase HI. The C-terminal seven residues are in cyan.

CD spectra of the wild-type, C58/145A, and ΔC6 Sto-RNase HI were measured in the far-UV region to examine the effect of the mutations on the overall secondary structure. As shown in Figure 2A, the shape of the spectra was almost the same for the wild-type and variant proteins. The crystal structure of ΔC6 Sto-RNase HI was solved at a resolution of 1.66 Å, as shown in Figure 2B. Two molecules are contained per asymmetric unit. The root-mean-square deviations of the Cα atoms for the A and B chains of the ΔC6 variant against the wild-type protein were 0.446 and 0.436Å. These results showed that the overall structures of both C58/145A and ΔC6 Sto-RNase HI resembled the wild-type protein. C-terminal residues 142 and 143 of ΔC6 Sto-RNase HI were not observed because of disorder, indicating that deletion of the previous C-terminal residues made the new C-terminus flexible.

### Stability of Sto-RNase HI variants

**Figure 3:**
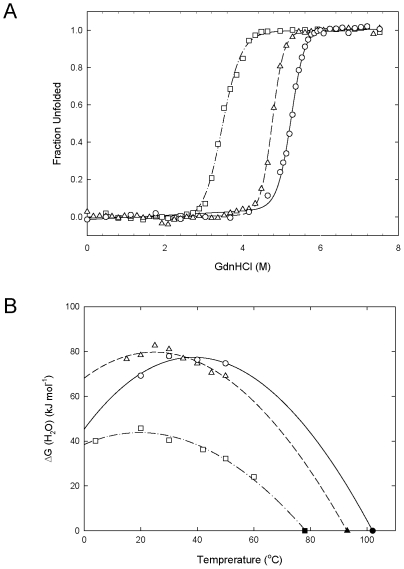
GdnHCl-induced equilibrium unfolding curves and thermodynamic stability profiles (temperature dependence of ΔG(H2O)) of Sto-RNase HI. Wild-type (solid line and circles), C58/145A (dashed line and triangles), and ΔC6 (dot-dashed line and squares). (A) GdnHCl-induced equilibrium unfolding at 20°C. The apparent fraction of unfolded protein is shown as a function of GdnHCl concentration. Lines are best fits to a two-state equation. (B) Thermodynamic stability profiles. Closed symbols are the T_m_ value from the heat-induced unfolding experiment [14]. Lines represent the fit of Eq. (3) using both equilibrium and heat-induced unfolding data.

Heat-induced unfolding of the wild-type, C58/145A, and ΔC6 Sto-RNase HI variants was previously analyzed by differential scanning calorimetry (DSC) at pH 3.0 [14]. The denaturation temperature is 102°C for the wild-type, 93°C for C58/145A and 78°C for ΔC6 Sto-RNase HI. These results indicated that Sto-RNase HI is a hyperthermostable protein with a denaturation temperature beyond the boiling temperature, and is destabilized by 9°C by elimination of the disulfide bond and by 24°C by C-terminal truncation. In this work, we confirmed stability changes in the variant proteins using GdnHCl-induced equilibrium unfolding experiments at various temperatures (Figure 3A). GdnHCl denaturation was reversible under all conditions examined. The ΔG(H_2_O) value at each temperature was calculated, and the resultant values are plotted as a function of temperature for a stability profile in Figure 3B. When fitting these values to Eq. (3), the T_m_ value, which is the thermal denaturation temperature obtained from the heat-induced unfolding experiment [14], was used (ΔG(H_2_O) = 0 at T_m_). The thermodynamic parameters are in Table 2. For C58/145A Sto-RNase HI, the curve shifts towards a lower temperature, indicating that destabilization is caused by entropic penalty [24]. This suggests that elimination of the disulfide bond mainly affected the conformation of C58/145A Sto-RNase HI in the denatured state. In contrast, the ΔC6 variant shifted the curve downward and flattened it. This is the result of decreases in ΔH and ΔC_p_. Because the C-terminal truncation eliminates hydrogen bonds and hydrophobic interactions, this result suggested that elimination of these forces at the C-terminal region was mainly responsible for the decreases in ΔH and ΔC_p_, resulting in the destabilization of ΔC6 Sto-RNase HI. We concluded that the C-terminal residues of Sto-RNase HI contributed to stability through local interactions.

**Table 2:** Thermodynamic parameters for denaturation of wild-type, C58/145A and ΔC6 Sto-RNase HI.

	T_m_ (°C)	ΔH(T_m_) (kJ mol^−1^)	ΔC_p_ (kJ mol^−1^ K^−1^)
Wild-type	102^a^	890±21^b^	12.8±0.6^b^
C58/145A	87.3^a^	827±24^b^	11.0±0.7^b^
ΔC6	79.1^a^	508±21^b^	7.9±0.7^b^

a Data from [14].

b Errors are standard error values from the data fitting using Eq. (3).

### Unfolding kinetics of Sto-RNase HI variants

**Figure 4:**
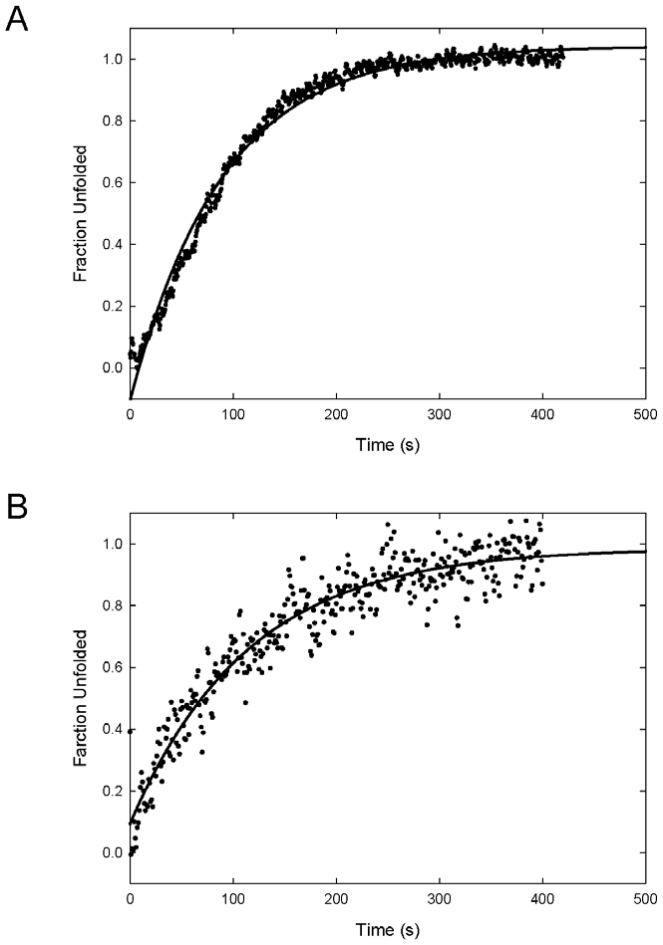
GdnHCl-induced kinetic unfolding curves of Sto-RNase HI. Lines represent the fit of Eq. (4). (A) C58/145A. Curve represents the unfolding trace to a final concentration of 5.8 M GdnHCl. (B) ΔC6. Curve represents the unfolding trace to a final concentration of 5.0 M GdnHCl.

Sto-RNase HI is highly stable, as indicated by its remarkably slow unfolding [14], [15]. To understand the stabilization mechanism of the C-terminus of Sto-RNase HI, we performed GdnHCl-induced kinetic unfolding of the variant proteins at 25°C (Figures 4A and 4B). The reaction was initiated by jumps to various GdnHCl concentrations followed by CD measurements. All kinetic traces were described by a single exponential. We calculated k_u_(H_2_O), which is the rate constant for unfolding in the absence of GdnHCl, from the GdnHCl concentration dependence of the logarithms of the apparent unfolding rate constant (k_app_), which is the linear correlation of ln k_app_ with GdnHCl concentration. The k_u_(H_2_O) was 5.7×10^−11^ s^−1^ for the wild-type, 1.0×10^−6^ s^−1^ for C58/145A, and 1.7×10^−5^ s^−1^ for ΔC6 Sto-RNase HI. Both variant proteins unfolded much faster than the wild-type protein. These results suggested that the C-terminal residues of Sto-RNase HI also contribute to the slow unfolding of this protein through hydrophobic interactions, because hydrophobic effects are one reason for the slow unfolding of ribonuclease HII from hyperthermophilic archaeon *Thermococcus kodakaraensis* [25], [26].

### Attachment of the C-terminal residues of Sto-RNase HI to other proteins

**Figure 5:**
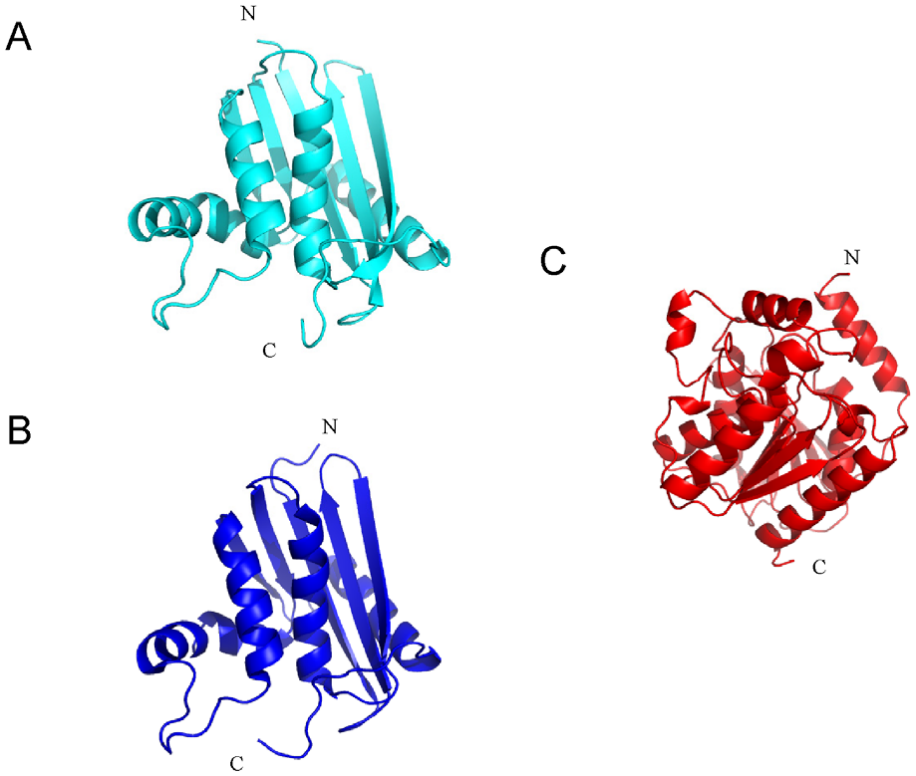
Crystal structure of So-RNase HI, Ec-RNase HI, and Sto-esterase. (A) So-RNase HI, (B) Ec-RNase HI and (C) Sto-esterase.

As described above, the C-terminal residues of Sto-RNase HI contribute to stability through local hydrogen bonds, hydrophobic interactions, and a disulfide bond. This suggests the possibility of their use as a stabilization tag, because they structurally affect only their local region, but thermodynamically affect overall stability. We tested the effect on stability of attaching the C-terminal residues of Sto-RNase HI to So-RNase HI, Ec-RNase HI, and Sto-esterase. So-RNase HI and Ec-RNase HI are homologous to Sto-RNase HI (amino acid sequence identity of 19 and 18% to Sto-RNase HI) but lacking a C-terminal anchoring (Figures 5A and 5B), so a positive effect on stabilization was expected. Since So-RNase HI is from a psychrotrophic bacterium, it may be particularly easy to stabilize. In contrast, Sto-esterase (Figure 5C) is a hyperthermophilic protein and non-homologous with Sto-RNase HI, and might be difficult to stabilize.

**Figure 6:**
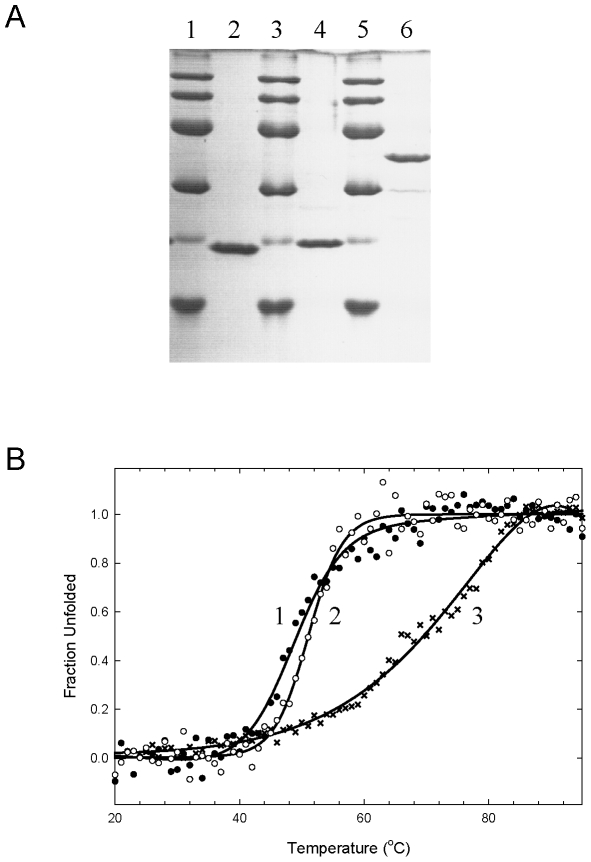
SDS-PAGE and heat-induced unfolding curves of chimeric proteins. (A) SDS-PAGE. Lanes 1, 3, 5, are a low-molecular weight marker kit (GE Healthcare). Lanes 2, 4, 6 are purified chimeric So-RNase HI, Ec-RNase HI and Sto-esterase. (B) Heat-induced unfolding. The apparent fraction of unfolded protein is shown as a function of temperature. Curves 1, 2 and 3 represent the unfolding traces of chimeric So-RNase HI (closed circles), Ec-RNase HI (open circles) and Sto-esterase (cross). Lines are best fits to a two-state equation.

We designed chimeric proteins with the C-terminal seven residues (IGCIILT) of Sto-RNase HI fused to the original C-terminal of So-RNase HI, Ec-RNase HI, or Sto-esterase. Overproduction and purification of the chimeric proteins was carried out as for the wild-type proteins, as shown in Figure 6A. Although the attached residues were somewhat hydrophobic, the proteins did not aggregate from the decrease in solubility. The heat-induced unfolding curves of the chimeric proteins are depicted in Figure 6B. Denaturation temperatures are in Table 3. The results showed the tag stabilized all proteins including the hyperthermophilic Sto-esterase. This indicated that stabilization tag was effective at stabilizing proteins.

**Table 3:** Denaturation temperatures of chimeric proteins.

	T_m_ (°C)	T_m_(wild-type) (°C)	ΔT_m_ (°C)
So-RNase HI	49.1^a^	30.4^b^	+18.7
Ec-RNase HI	52.2^a^	49.9^c^	+1.3
Sto-esterase	72.4^a^	67.9^a^	+4.5

a Errors are ±0.3°C.

b Data from [16].

c Data from [28].

The three chimeric proteins were stabilized, but to different degrees between 18.7 and 1.3°C. This was the result of blind design without structural information. Especially, the effect was different between So-RNase HI and Ec-RNase HI, although Ec-RNase HI shows high amino-acid sequence identity (67%) to So-RNase HI. Since So-RNase HI and Ec-RNase HI are homologous to Sto-RNase HI but do not have a corresponding cysteine residue to C58 of Sto-RNase HI, forming a new disulfide bond by attachment of the C-terminal was not expected. So-RNase HI was stabilized the most, suggesting positive interactions by hydrophobic effect and hydrogen bonds through the tag. In contrast, Ec-RNase HI appeared to fail in C-terminal anchoring. These results suggest that C-terminal elongation may bring about an effect beyond expectation. Stabilization mechanism of the chimeric proteins will be revealed by the structural determination and detailed thermodynamic analysis in future.

Random elongation of C-terminal residues often stabilizes proteins, and deletion of C- or N-terminal residues is often destabilizing [27]–[29]. The overall structure of proteins is not usually affected by fusion of peptides to the C-terminal region [30], [31]. Recently, the C-terminal region has been reported as important for folding and stability of staphylococcal nuclease and onconase [32], [33]. These results suggest a strong likelihood of protein stabilization by a C-terminal tag.

In conclusion, we showed the validity of a stabilization tag using the C-terminal residues of Sto-RNase HI. A stabilization tag could be easy to use because genes can be modified without structural information about the proteins they encode. We do not suggest that the C-terminal residues used here are the best stabilization tag. A universal stabilization tag may exist that stabilizes all proteins, or an optimal stabilization tag could be designed for individual proteins.
